# Retraction Statement: Predictive validity of a medication adherence measure in an outpatient setting

**DOI:** 10.1111/jch.14718

**Published:** 2023-08-18

## Abstract

Morisky, D.E., Ang, A., Krousel‐Wood, M. and Ward, H.J. (2008), Predictive Validity of a Medication Adherence Measure in an Outpatient Setting. The Journal of Clinical Hypertension, 10: 348–354. https://doi.org/10.1111/j.1751‐7176.2008.07572.x.

The above article, published online on May 02, 2008 on Wiley Online Library (wileyonlinelibrary.com), has been retracted by agreement between the journal's Editor‐in‐Chief, Dr. Ji‐Guang Wang, and Wiley Periodicals LLC. Following publication, concerns were raised by a third party regarding the statistical analysis presented in the article. The Journal conducted an independent statistical review of the article and concluded that the results were misleading due to issues regarding the sensitivity and specificity of the medical adherence scale used. The authors responded to the Journal's request to address the findings of the independent statistical review, but were unable to adequately address the concerns. As a result, the Journal no longer has confidence in the reported conclusions and is issuing this retraction.

Morisky, D.E., Ang, A., Krousel‐Wood, M. and Ward, H.J. (2008), Predictive Validity of a Medication Adherence Measure in an Outpatient Setting. The Journal of Clinical Hypertension, 10: 348–354. https://doi.org/10.1111/j.1751‐7176.2008.07572.x.

The above article, published online on May 02, 2008 on Wiley Online Library (wileyonlinelibrary.com), has been retracted by agreement between the journal's Editor‐in‐Chief, Dr. Ji‐Guang Wang, and Wiley Periodicals LLC. Following publication, concerns were raised by a third party regarding the statistical analysis presented in the article. The Journal conducted an independent statistical review of the article and concluded that the results were misleading due to issues regarding the sensitivity and specificity of the medical adherence scale used. The authors responded to the Journal's request to address the findings of the independent statistical review, but were unable to adequately address the concerns. As a result, the Journal no longer has confidence in the reported conclusions and is issuing this retraction.

